# Intrinsic capacity decline and self-rated health in patients ≥ 65 years: a survey in general practice waiting rooms in Norway

**DOI:** 10.1080/02813432.2026.2748805

**Published:** 2026-10-27

**Authors:** Isabel Sebjørnsen, Marit Stordal Bakken, Stefán Hjörleifsson, Øystein Hetlevik, Gunnar Tschudi Bondevik, Camilla Berg-Utby, Trygve Skonnord, Anja Maria Brænd, Maria Romøren

**Affiliations:** ^a^ National Centre for Emergency Primary Health Care, NORCE Research, Bergen, Norway; ^b^ Department of Clinical Science, University of Bergen, Bergen, Norway; ^c^ Department of Medicine, Haraldsplass Deaconess Hospital, Bergen, Norway; ^d^ Section for General Practice, Department of Global Public Health and Primary Care, University of Bergen, Bergen, Norway; ^e^ Østre Agder Inter-municipal Cooperation, Agder, Norway; ^f^ Department of General Practice, Institute of Health and Society, University of Oslo, Oslo, Norway; ^g^ General Practice Research Unit, Institute of Health and Society, University of Oslo, Oslo, Norway

**Keywords:** Intrinsic capacity, functional ability, integrated care, older adults, general practice, Health Status, Geriatric Assessment, Activities of Daily Living, Aged, General Practice, Primary Health Care

## Abstract

**Objectives:**

To examine the prevalence of, and factors associated with, intrinsic capacity (IC) decline among older adults in Norwegian general practice. Second, to assess how IC decline relates to self-rated health (SRH).

**Design and setting:**

Survey examination in general practice waiting rooms in Norway in 2025.

**Participants:**

Adults aged ≥ 65 years.

**Measurements:**

IC decline within six domains (cognition, locomotion, vitality, vision, hearing and psychological well-being) was assessed using a modified version of the Integrated Care for Older People (ICOPE) Step 1 screening tool. SRH was recorded on a Likert scale. Poisson regression was used to examine associations between demographic covariates and IC decline, and between IC decline and poor SRH.

**Results:**

The study included 2154 respondents (54% females); 44% aged 65–74, 44% aged 75–84 and 11% ≥ 85 years. The overall prevalence of IC decline was 60%. Hearing loss (31%), limited mobility (23%) and malnutrition (21%) were predominant. Across domains, decline was associated with higher age and financial difficulties. Individuals with decline in ≥ 1 domain(s) were more likely to report poor SRH (RR 4.51, 95% CI 3.05–6.67), and likelihood increased by number of affected domains. Depressive symptoms and limited mobility showed the strongest independent associations with poor SRH (RR 3.84, 95% CI 2.92–5.05 and RR 2.55, 95% CI 1.94–3.35, respectively).

**Conclusion:**

The IC approach appears relevant to Norwegian general practice. Our findings highlight important target areas for preserving function in a growing older population. Whether and how ICOPE can be integrated into Norwegian primary care requires further study.

## Introduction

In response to the growing older population, the World Health Organization (WHO) has launched the term *intrinsic capacity* as a holistic measure of healthy ageing [1]. The term refers to *the physical and mental capacities that an individual can draw on to live independently and thrive*. An individual’s functional abilities, and consequently his or her ability to live independently, is a product of the interaction between intrinsic capacity and the environment [2]. Thus, decline in intrinsic capacity manifests as loss of functional abilities. Progressive loss of intrinsic capacity can lead to frailty, a state of increased vulnerability to illness and adverse health outcomes. Frailty is strongly associated with extensive health care utilisation [3]. However, there is strong evidence that the onset of frailty can be delayed through early and targeted interventions [4,5].

Substantial efforts have been devoted to developing methods for identifying frailty, leading to widespread adoption of tools such as Fried’s criteria, Clinical Frailty Scale and Frailty Index [6]. The concept of intrinsic capacity should be seen as complementary to addressing frailty in the ageing population [7]. While frailty assessment is likely to retain its role in risk stratification and for supporting clinical decision-making throughout the ageing trajectory, the value of assessing intrinsic capacity lies in earlier, preventive stages of care, where maintaining function and delaying frailty are realistic and meaningful goals [1,7,8].

To promote healthy ageing and aid in the preservation of functional independence, WHO has launched the Integrated Care for Older People (ICOPE) framework [9]. The purpose of ICOPE is to enable optimisation of functional abilities through early detection of, and interventions for, decline in intrinsic capacity. The framework is explicitly designed for integration in primary health care. Six core domains central to maintaining independence are targeted: cognition, locomotion, vitality, vision, hearing and psychological well-being. The first step is screening for potential decline in intrinsic capacity within these six domains, thus providing an overview of the patient’s capabilities and potential intervention areas. Subsequent steps involve confirming impairment with in-depth tests, assessing underlying causes and creating a personalised plan to support functional ability. The framework links identification of decline to person-centered care pathways and evidence-based interventions tailored to each specific domain. Recommended interventions include exercise programmes and fall prevention strategies, nutritional support, management of sensory impairments, psychological therapies, as well as treatment of risk factors and underlying conditions, medication review and optimisation, and interventions to promote cognitive function and social participation. Among these, multicomponent exercise programmes have shown particularly promising results and are increasingly recognised as a key intervention for promoting healthy ageing [10].

Internationally, there is growing interest in intrinsic capacity and the ICOPE framework. However, studies within the field have so far been conducted in countries differing greatly from the Nordic in terms of population health, health care organisation and/or social support structures [11–13]. In Norway, general practitioners (GPs) have a central role in the primary care system, and are uniquely placed to identify, address and follow up age-related changes in function [12,14]. Knowledge of how intrinsic capacity is distributed across domains and demographic groups among older adults attending GP practices may help inform the relevance and potential application of the ICOPE framework in Norway. Furthermore, it may be useful to know how the domains prioritised within the framework correspond to aspects of health that matter to older adults in this setting. Self-rated health provides a useful perspective in this regard, as it reflects the patient’s own perception of overall health and well-being. Poor self-rated health is a well-established marker of vulnerability associated with mortality, morbidity and decreased quality of life [15]. In this study, poor self-rated health was used to explore the relevance of intrinsic capacity from the perspective of older adults.

### Aim

The aim of this study was to examine the prevalence of, and factors associated with, self-reported decline in six domains of intrinsic capacity among older patients attending Norwegian GP practices. Moreover, to assess the association between decline in intrinsic capacity and poor self-rated health in this population.

## Material and methods

### Study design

This was an observational questionnaire-based study conducted in waiting rooms of GP practices in Norway in 2025. The study was conducted within the *Felles allmennmedisinsk forskning i Norge* (FAMFIN) project, a collaborative initiative between the general practice research groups at the University of Bergen (UiB) and the University of Oslo (UiO). FAMFIN is structured as a semi-annual questionnaire survey embedded in the six-week general practice placement during the final year (year 6) of the undergraduate medical curriculum of UiB and year 5 of the UiO curriculum. The students are assigned the task of recruiting patients for participation in a survey, with alternating themes each semester.

The survey for the current study was developed by a team with representatives from primary care and geriatric medicine. A draft was piloted among seven individuals aged 75 years and older prior to the data collection, and adjustments were made in accordance with their feedback. The final version of the survey consisted of half an A4-page of information about the project and what participation entailed, followed by one and a half page of survey items. The survey was available as a paper-based survey only.

### Setting

In Norway, nearly all inhabitants are registered with a named GP responsible for providing medical care, coordinating services, and acting as gatekeeper to specialised care [16,17]. A typical GP practice consists of three to six physicians working together [18]. On average, each GP provide care for 978 patients (2024) [19]. Among residents aged 65 and older, 90% have at least one contact with their GP each year, and consultation rates increase with age [20]. Individuals residing in nursing homes are still formally registered with the GP, but in practice, medical responsibility is transferred to the attending physician at the nursing home [21].

Each semester, approximately 200 medical students affiliated with UiB and UiO complete placements in roughly 175 GP practices in Southern Norway. The exact number of contributing practices could not be determined, as multiple students were placed at some practices and not all participated.

### Participants

This study targeted patients aged 65 years and older attending the participating GP practices. Proficiency in Norwegian language was a prerequisite for participation, as the survey was only available in Norwegian.

### Data collection

Data were collected during the spring (March – May) and autumn (September – December) semesters of 2025. Each student was instructed to invite 20 consecutive patients for inclusion. Study information was provided to eligible patients orally by the medical students, and in written form embedded in the survey and through posters in the waiting rooms. Patients who were willing to participate, completed the survey while waiting for their consultation and returned it in a sealed envelope to the receptionist. The survey was answered anonymously, and no patients could be identified after submission.

### Variables

#### Demographics

Demographic data included sex (response options: *female* and *male*), age group (*65–69*, *70–74*, *75–79*, *80–84*, *≥ 85 years*), highest completed education (*none*, *primary school*, *high school*, *higher education*) and birthplace (*Norway*, *other European country*, *rest of the world*). In addition, information about the respondents’ financial circumstances were collected through asking “How is it for you to make ends meet financially?” on a 5-level Likert scale (*easy, fairly easy, neither easy nor difficult, fairly difficult, difficult*).

Due to low cell counts in some response categories, and to enhance the interpretability of the results, selected categories were combined for the analyses:

- Patients were arranged in three groups by age: *65–74*, *75–84*, *≥ 85 years*
- For education, the response categories *none* and *primary school* were pooled: *none/primary school*
- For birthplace, *other European country* and *rest of the world* were pooled: *other country*
- For the economy variable, the response categories *fairly difficult* and *difficult* were pooled: *difficult*

#### Intrinsic capacity

The screening tool that constitutes Step 1 of the ICOPE framework has been translated to Norwegian by Hollister et al. (ICOPE-n) [22]. The screening consists of simple tests and questions and is designed so that it can be conducted by health care professionals, the older adults themselves (self-assessment) or next-of-kins [9]. Our assessment of decline in intrinsic capacity was based on the ICOPE-n; however, to fit the context of data collection in waiting rooms, we omitted tests and relied exclusively on self-reported questions. The items identified *potential* decline within the six intrinsic capacity domains: cognitive decline, limited mobility, malnutrition, visual impairment, hearing loss, and depressive symptoms. The items included in the survey can be viewed in Table 1. In subsequent sections, findings are referred to as ‘decline in intrinsic capacity’ for simplicity.

**Table 1.** Assessment for intrinsic capacity based on the integrated care for older people (ICOPE) framework, as included in the survey of adults ≥ 65 years in waiting rooms of Norwegian GP practices (2025).

Domain	Decline	Item(s)	Response options
Cognition	Cognitive decline	Do you have any problems with memory or orientation (such as knowing where you are or what time it is)?	**Yes** | No
Locomotion	Limited mobility	Do you think you would be able to stand up and sit down from a chair five times in 14 s, without using your arms?	Yes | **No**
Vitality	Malnutrition	Have you	
		unintentionally lost more than 3 kg over the last three months?	**Yes** | No
		experienced loss of appetite?	**Yes** | No
Vision	Visual impairment	Do you have any problems with your vision: difficulties in seeing far or reading, despite using glasses?	**Yes** | No
Hearing	Hearing loss	Are you able to hear whispers at an armlengths’ distance?	Yes | **No**
Psychological well-being	Depressive symptoms	Over the past two weeks, have you been bothered by	
		feeling down, depressed or hopeless?	Not at all | Several days | **More than half the days | Nearly every day**
		little interest or pleasure in doing things?	Not at all | Several days | **More than half the days | Nearly every day**

*Response options in bold indicate decline in intrinsic capacity domain*.

*Adapted from: Integrated care for older people (ICOPE). Guidance for person-centred assessment and pathways in primary care. 2nd ed. Geneva: World Health Organization; 2024.*

#### Self-rated health

Self-rated health compared to others of same age was registered on a 5-level Likert scale with response options *very good*, *fairly good, neither good nor poor*, *fairly poor* and *very poor.* For analyses, we pooled the two latter categories to one *poor self-rated health* category.

### Outcomes

The primary outcomes were:

- overall and domain-specific prevalence of decline in intrinsic capacity
- factors associated with decline in intrinsic capacity

Secondary outcomes included associations between overall and domain-specific decline in intrinsic capacity and poor self-rated health.

### Statistical analysis

Patient characteristics are described by numbers and percentages. Any intrinsic capacity decline reported within each domain was given a score of 1, otherwise 0. For each patient, the sum of domains with decline in intrinsic capacity was calculated by summarising the score of the six domains, yielding a score between 0 (preserved intrinsic capacity across all six domains) and 6 (decline in intrinsic capacity across all six domains). Overall prevalence of decline in intrinsic capacity is reported as the percentage of respondents with scores ≥ 1. The number of domains with decline in intrinsic capacity is presented by the median score (interquartile range (IQR)), along with the distribution. Domain-specific prevalence of intrinsic capacity decline is reported as percentages of respondents with score 1 within each domain.

Bivariate analyses were conducted to explore unadjusted associations between intrinsic capacity decline, demographic variables (age, sex, birthplace, education and economy), and self-rated health. To illustrate unadjusted patterns, descriptive plots are included, showing:

- the prevalence of decline across the six intrinsic capacity domains by demographic characteristics (bar charts)
- distribution across number of domains with decline and relationship with poor self-rated health (bar-line chart)

Multivariable Poisson regression models with robust standard errors were used to assess which demographic variables were independently associated with decline in intrinsic capacity. Age group *65–74 years*, *female* sex, *none/primary school* as highest completed education, birthplace *Norway* and response option *easy* for the economy item were used as reference groups.

Associations between decline in intrinsic capacity within each domain and poor self-rated health were also examined using Poisson regression models with robust standard errors. The reference groups for these analyses were individuals with no decline (any or within the respective domains). We present results from two models:

Model 1: Adjusted for demographic covariates (sex, age, highest completed education, financial circumstances and birthplace)

Model 2: Adjusted for scores in the other domains of intrinsic capacity and demographic covariates (sex, age, highest completed education, financial circumstances and birthplace)

We also performed subgroup analyses, with stratification by age and sex instead of adjustment, to explore potential age- and sex-specific patterns in risk of poor self-rated health across domains.

Participants with missing values were omitted from analyses. Hence, prevalences indicate participants with decline in intrinsic capacity among patients who responded to the respective items. Associations are reported as relative risk (RR) with 95% confidence interval (CI). STATA version 19.5 was used for statistical analyses (StataCorp, USA) and significance level was set at α = 0.05.

### Ethics

The study was conducted in accordance with the Helsinki Declaration and approved by the Regional Committee for Medical and Health Research Ethics, South-East Norway (REK Sør-Øst; reference number 815288, approved 11/2024). Participation was voluntary and based on consent, provided implicitly by the patients through completing and submitting the survey.

## Results

### Patient characteristics

A total of 2154 individuals responded to the survey, a response rate of 77%. Among the respondents, 954 (44%) were between 65 and 74 years, 944 (44%) between 75 and 84 years and 246 (11%) ≥ 85 years. The majority were female (54%). Educational levels were high, with 823 (39%) responding to have completed higher education. Patient characteristics are further described in Table 2.

**Table 2.** Characteristics of individuals included in the survey examination of adults ≥ 65 years in waiting rooms of Norwegian GP practices (2025) (*N* = 2154).

	n	%
**Age (years)**		
65–74	954	44
75–84	944	44
≥ 85	246	11
*Missing*	*10*	
**Sex**		
Female	1135	54
Male	950	46
*Missing*	*69*	
**Highest completed education**		
None/primary school	483	23
High school	794	38
Higher education	823	39
*Missing*	*54*	
**Birthplace**		
Norway	2056	96
Other country	86	4
*Missing*	*12*	
**Economy – how is it to make ends meet?**		
Easy	752	35
Fairly easy	668	31
Neither easy nor difficult	618	29
Difficult	106	5
*Missing*	*10*	

### Prevalence of intrinsic capacity decline

Data for calculation of overall prevalence and number of domains with decline were available from 2122 respondents. Of these, 1269 (60%) reported decline in one or more intrinsic capacity domain(s). The median number of domains with decline was 1 (IQR 0–2). The number of patients with decline in one domain was 650 (31%), 342 (16%) reported decline in two domains, 172 (8%) in three domains, 70 (3%) in four domains, 29 (1%) in five domains and 6 (0%) had decline across all six domains.

Table 3 shows the number and percentage of respondents reporting decline within each domain. Due to missing responses, the number of participants included in these analyses (N) varied across domains and is reported in the table. Hearing loss (31%), limited mobility (23%) and malnutrition (21%) were the three predominant manifestations of intrinsic capacity decline in the study population.

**Table 3.** Prevalence of domain-specific intrinsic capacity decline among individuals included in the survey examination of adults ≥ 65 years in waiting rooms of Norwegian GP practices (2025) (*N* = 2154).

	n/N	%
Cognitive decline	132/2108	6
Limited mobility	484/2094	23
Malnutrition	453/2107	21
Visual impairment	376/2097	18
Hearing loss	658/2103	31
Depressive symptoms	208/2095	10

*The number of respondents varied across domains due to missing responses. Accordingly, n denotes the number of participants reporting decline within a given domain, and N the number of participants with available data for that specific domain*.

### Factors associated with intrinsic capacity decline

#### Unadjusted associations

Figure 1 illustrates the percentage of patients with decline in intrinsic capacity within each of the domains by age, sex, highest completed education, birthplace and economy. As shown, the proportion reporting decline increased with age, decreased with increasing educational level and was higher among patients born outside Norway and those reporting financial difficulties, across all domains. Only minimal differences were observed between male and female respondents.

**Figure 1.**
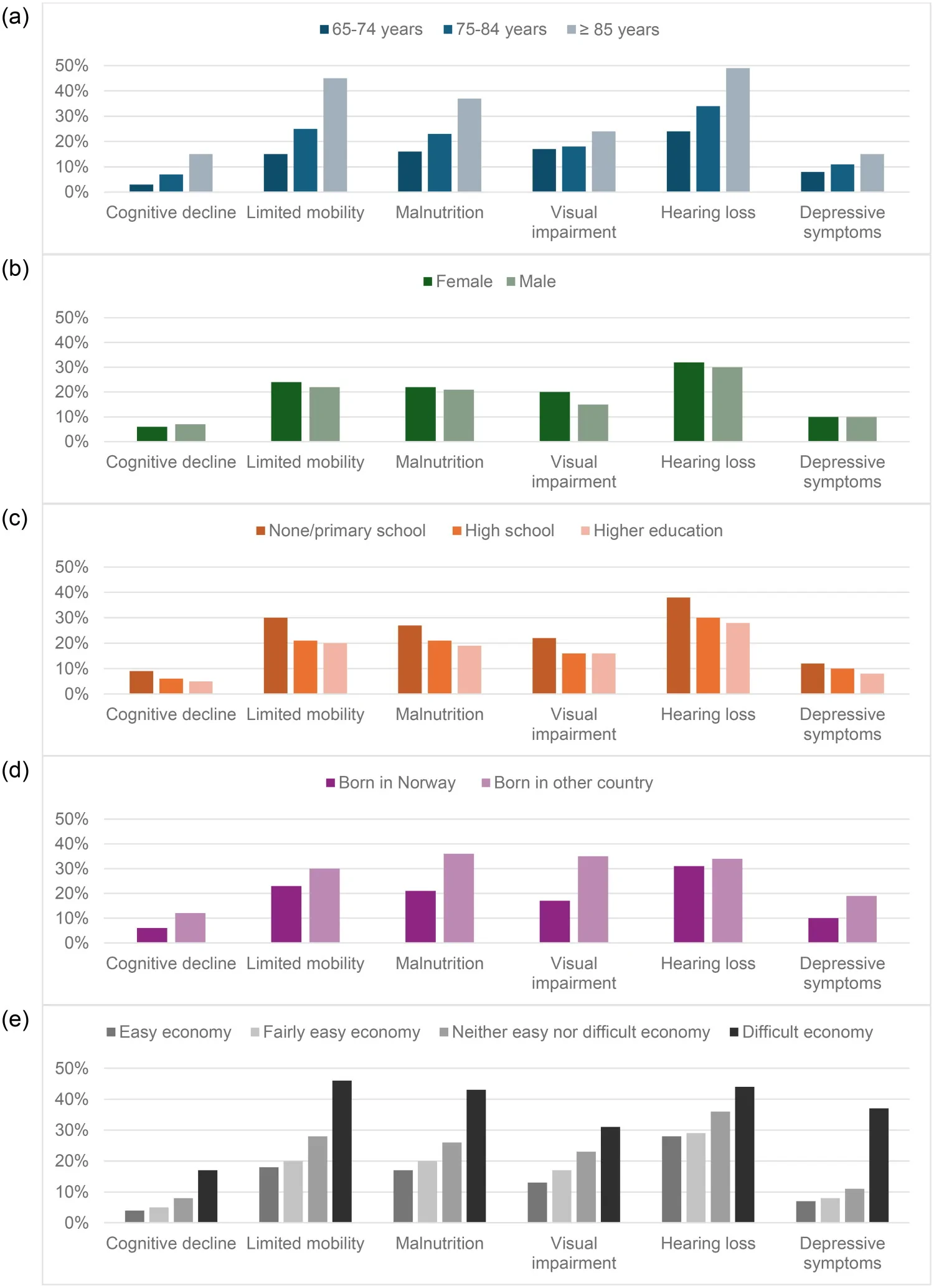
Proportions of self-reported intrinsic capacity decline across ICOPE domains by demographic variables: (a) age, (b) sex, (c) highest completed education, (d) birthplace and (e) economy. The study population consists of older adults recruited in waiting rooms of Norwegian GP practices in 2025.

A dose-response relationship was found between numbers of domains showing decline and age. In the youngest age group, no decline or decline in only one domain dominated (79%). Among patients ≥ 85 years, close to one third reported decline in three or more domains. Similar patterns were seen for the economy and education variables; decreasing proportions reporting no decline or decline in only one domain with increasing financial difficulties and decreasing level of education completed. Among Norwegian-born individuals, 72% reported no decline or decline in one domain only, whereas the same was the case for 53% of individuals born abroad. Only small differences were observed for number of domains with decline between the sexes.

#### Adjusted associations

The relative risk of intrinsic capacity decline in one or more domain(s) was significantly higher in older patients (RR 1.26, 95% CI 1.16–1.37 for *75–84* years; RR 1.65, 95% CI 1.51–1.81 for *≥ 85 years*). It was also higher in patients born abroad (RR 1.33, 95% CI 1.18–1.50) and patients reporting financial difficulties (RR 1.57, 95% CI 1.40–1.76 for *difficult to make ends meet*). Higher education was associated with lower risk of any decline in intrinsic capacity (RR 0.89, 95% CI 0.81–0.97).

Increasing age and financial difficulties were linked to decline in intrinsic capacity across all domains also in the adjusted analyses (Table 4). Visual impairment was the only domain significantly associated with sex (higher in females) after adjustment for other variables. Cognitive decline, limited mobility, malnutrition and visual impairment were associated with being born abroad. Limited mobility, malnutrition, visual impairment and hearing loss were associated with lower education levels.

**Table 4.** Adjusted relative risks for intrinsic capacity decline across domains by demographic variables among individuals included in the survey examination of adults ≥ 65 years in waiting rooms of Norwegian GP practices (2025) (*N* = 2154).

	Cognitive decline				Limited mobility				Malnutrition			
	n (%)	RR	95% CI	p-value	n (%)	RR	95% CI	p-value	n (%)	RR	95% CI	p-value
**Age (years)**												
65–74 (ref)	32 (3)	1.00			143 (15)	1.00			151 (16)	1.00		
75–84	62 (7)	**2.17**	**1.41-3.34**	**<0.001**	231 (25)	**1.67**	**1.37-2.02**	**<0.001**	211 (23)	**1.40**	**1.16-1.70**	**0.001**
≥85	37 (15)	**4.99**	**3.08-8.09**	**<0.001**	108 (45)	**3.15**	**2.55-3.89**	**<0.001**	89 (37)	**2.36**	**1.88-2.96**	**<0.001**
**Sex**												
Female (ref)	65 (6)	1.00			269 (24)	1.00			242 (22)	1.00		
Male	61 (7)	1.22	0.86-1.73	0.267	201 (22)	0.94	0.80-1.10	0.436	193 (21)	1.00	0.84-1.18	0.981
**Highest completed education**												
None/primary school (ref)	43 (9)	1.00			142 (30)	1.00			126 (27)	1.00		
High school	43 (6)	0.73	0.48-1.11	0.145	164 (21)	**0.80**	**0.66-0.97**	**0.023**	163 (21)	0.84	0.69-1.03	0.097
Higher education	42 (5)	0.66	0.43-1.02	0.064	158 (20)	**0.77**	**0.63-0.94**	**0.009**	151 (19)	**0.78**	**0.63-0.96**	**0.020**
**Birthplace**												
Norway (ref)	120 (6)	1.00			456 (23)	1.00			419 (21)	1.00		
Other country	10 (12)	**2.16**	**1.13-4.14**	**0.020**	26 (30)	**1.40**	**1.01-1.95**	**0.045**	31 (36)	**1.73**	**1.27-2.35**	**0.001**
**Economy – how is it to make ends meet?**												
Easy (ref)	31 (4)	1.00			133 (18)	1.00			123 (17)	1.00		
Fairly easy	35 (5)	1.17	0.72-1.89	0.530	131 (20)	1.13	0.90-1.41	0.285	129 (20)	1.22	0.97-1.53	0.092
Neither easy nor difficult	48 (8)	**1.79**	**1.14-2.80**	**0.011**	170 (28)	**1.57**	**1.28-1.93**	**<0.001**	155 (26)	**1.56**	**1.25-1.93**	**<0.001**
Difficult	18 (17)	**3.87**	**2.17-6.91**	**<0.001**	48 (46)	**2.59**	**1.98-3.39**	**<0.001**	45 (43)	**2.47**	**1.86-3.28**	**<0.001**
	Visual impairment				Hearing loss				Depressive symptoms			
	n (%)	RR	95% CI	p-value	n (%)	RR	95% CI	p-value	n (%)	RR	95% CI	p-value
**Age (years)**												
65–74 (ref)	158 (17)	1.00			227 (24)	1.00			74 (8)	1.00		
75–84	162 (18)	1.03	0.84-1.27	0.764	310 (34)	**1.40**	**1.21-1.63**	**<0.001**	97 (11)	**1.35**	**1.02-1.80**	**0.039**
≥85	56 (24)	**1.48**	**1.12-1.96**	**0.006**	118 (49)	**2.00**	**1.67-2.39**	**<0.001**	36 (15)	**1.98**	**1.35-2.91**	**<0.001**
**Sex**												
Female (ref)	220 (20)	1.00			351 (32)	1.00			112 (10)	1.00		
Male	140 (15)	**0.78**	**0.64-0.95**	**0.012**	283 (30)	1.01	0.89-1.15	0.842	89 (10)	1.03	0.79-1.35	0.806
**Highest completed education**												
None/primary school (ref)	103 (22)	1.00			177 (38)	1.00			57 (12)	1.00		
High school	126 (16)	**0.78**	**0.61-0.99**	**0.042**	232 (30)	**0.85**	**0.72-0.99**	**0.039**	78 (10)	0.87	0.63-1.21	0.415
Higher education	131 (16)	0.80	0.63-1.03	0.083	231 (28)	**0.80**	**0.68-0.94**	**0.008**	68 (8)	0.76	0.54-1.08	0.124
**Birthplace**												
Norway (ref)	343 (17)	1.00			626 (31)	1.00			191 (10)	1.00		
Other country	29 (35)	**2.00**	**1.43-2.80**	**<0.001**	28 (34)	1.14	0.83-1.57	0.419	16 (19)	1.62	1.00–2.63	0.051
**Economy – how is it to make ends meet?**												
Easy (ref)	98 (13)	1.00			208 (28)	1.00			54 (7)	1.00		
Fairly easy	109 (17)	1.30	1.00-1.69	0.051	189 (29)	1.04	0.87-1.23	0.681	49 (8)	0.95	0.65-1.39	0.797
Neither easy nor difficult	135 (23)	**1.69**	**1.31-2.18**	**<0.001**	214 (36)	**1.26**	**1.07-1.48**	**0.006**	65 (11)	1.39	0.98-1.98	0.068
Difficult	33 (31)	**2.02**	**1.38-2.95**	**<0.001**	46 (44)	**1.54**	**1.19-1.98**	**0.001**	39 (37)	**4.75**	**3.25-6.93**	**<0.001**

**Table 5.** Associations between decline in intrinsic capacity within each domain and poor self-rated health among older adults in waiting rooms of Norwegian GP practices (2025).

	Crude			Model 1			Model 2 (mutually adjusted)		
	RR	95% CI	p-value	RR	95% CI	p-value	RR	95% CI	p-value
Cognitive decline	**2.82**	**2.10-3.77**	**<0.001**	**2.28**	**1.63-3.19**	**<0.001**	1.23	0.87-1.73	0.235
Limited mobility	**3.51**	**2.79-4.41**	**<0.001**	**3.40**	**2.65-4.37**	**<0.001**	**2.55**	**1.94–3.35**	**<0.001**
Malnutrition	**2.23**	**1.77-2.82**	**<0.001**	**1.98**	**1.53-2.56**	**<0.001**	**1.31**	**1.00–1.71**	**0.048**
Hearing loss	**1.58**	**1.25-2.00**	**<0.001**	**1.47**	**1.15-1.88**	**0.002**	1.01	0.79-1.30	0.920
Visual impairment	**1.88**	**1.47-2.42**	**<0.001**	**1.70**	**1.30–2.21**	**<0.001**	1.17	0.90-1.53	0.240
Depressive symptoms	**5.95**	**4.82-7.36**	**<0.001**	**5.20**	**4.08-6.62**	**<0.001**	**3.84**	**2.92–5.05**	**<0.001**

*Relative risks (RRs) with 95% CIs and p-values from Poisson regression analyses. Model 1 is adjusted for demographic covariates (age, sex, birthplace, education and economy). Model 2 included all intrinsic capacity decline variables simultaneously and is adjusted for demographic covariates*.

### Associations between intrinsic capacity decline and poor self-rated health

#### Overall

The proportion of patients rating their health as poor compared to others their age increased with the number of domains showing decline (Figure 2).

**Figure 2.**
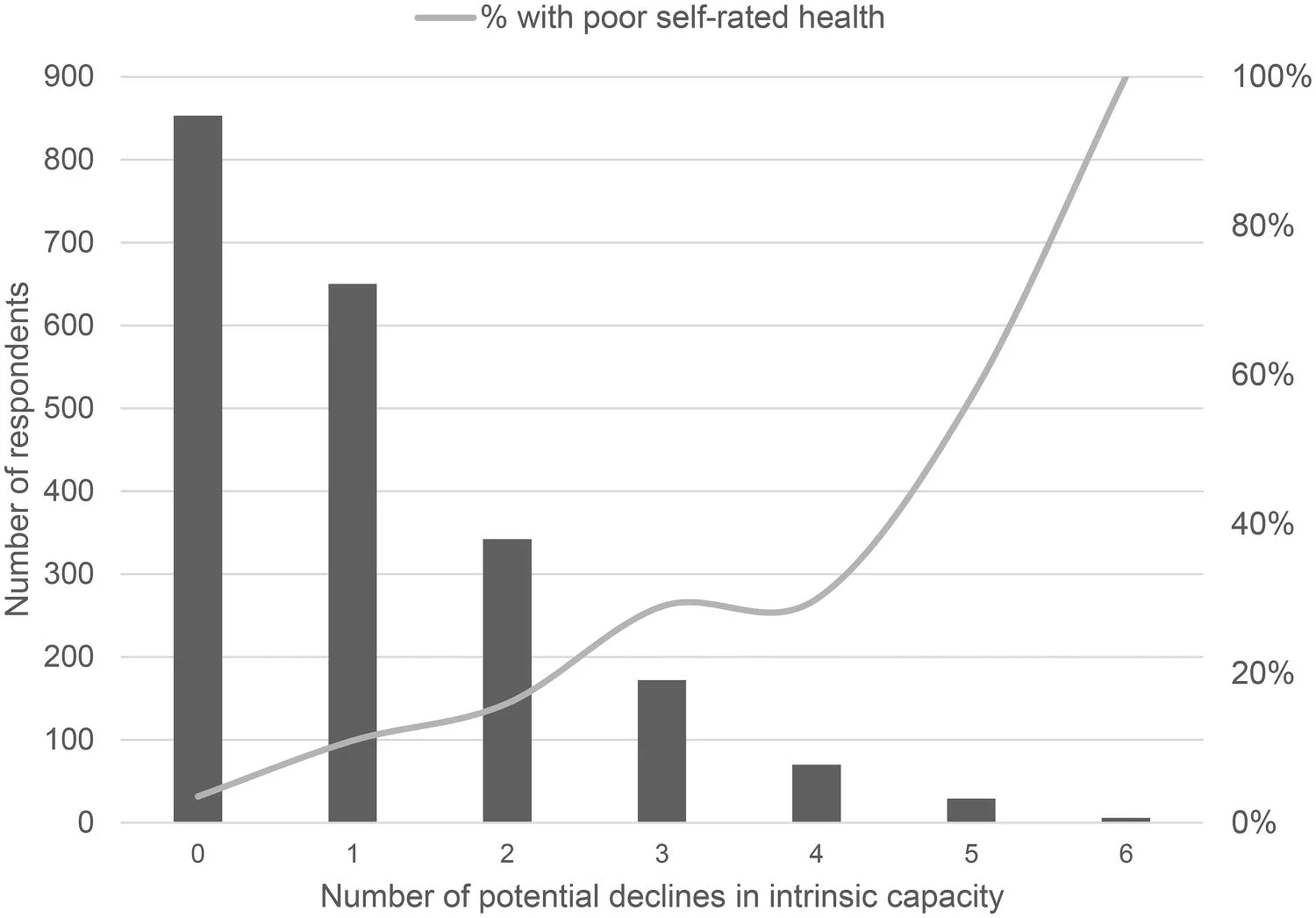
Number of patients (bars, left y-axis) by number of intrinsic capacity domains with self-reported decline (x-axis), with corresponding proportions reporting poor self-rated health (line, right y-axis). The study population consists of older adults recruited in waiting rooms of Norwegian GP practices in 2025.

This association remained after adjustment for demographic covariates: Individuals with decline in one or more intrinsic capacity domain(s) were more likely to report poor self-rated health compared to individuals with preserved intrinsic capacity (RR 4.51, 95% CI 3.05–6.67) and the relative risk increased from 3.15 (95% CI 2.06–4.82) for decline in one domain to 22.73 (95% CI 12.97–39.81) for decline in all six domains.

#### Domain-specific

In Model 1 (adjusted for demographic covariates only), intrinsic capacity decline was significantly associated with poor self-rated health across all domains (Table 5). In Model 2 (adjusted for demographic covariates *and* score in the other domains), only depressive symptoms (RR 3.84, 95% CI 2.92–5.05), limited mobility (RR 2.55, 95% CI 1.94–3.35) and malnutrition (RR 1.31, 95% CI 1.00–1.71) remained independently associated with poor self-rated health. Responses to the self-rated health item in patients with and without decline across domains are shown in detail in Supplementary file 1.

#### Age- and sex-stratified analyses

Stratified analyses suggested that the association between malnutrition, hearing loss and visual impairment and poor self-rated health became stronger with increasing age (Supplementary File 1). Conversely, the associations for cognitive decline and depressive symptoms appeared to weaken with higher age. The associations between cognitive decline and poor self-rated health, as well as between malnutrition and poor self-rated health, appeared somewhat stronger among males than females. In contrast, the associations for limited mobility and depressive symptoms appeared stronger among females than among males. However, across all age- and sex-stratified analyses, CIs were overlapping.

## Discussion

### Main findings

In this population of general practice patients aged 65 and older, more than half reported decline in intrinsic capacity in one or more domains. Hearing (31%), locomotion (23%) and vitality (21%) were the domains for which decline was most prevalent. Higher age and financial difficulties were associated with increased risk of decline across all six domains. There was a strong and dose-dependent association between reporting intrinsic capacity decline and rating one’s health as poor. Depressive symptoms and limited mobility showed particularly strong associations with poor self-rated health.

### Interpretation

Decline in intrinsic capacity was common across several domains and associated with poorer self-rated health. These findings suggest that intrinsic capacity is a relevant concept that captures dimensions of ageing that are important to older adults attending Norwegian general practices. Locomotion seems to be of particular significance, as decline within this domain was both among the most prevalent and associated with a two-and-a-half-fold higher risk of rating one’s health as poor, independent of intrinsic capacity scores within other domains.

Notably, our findings may be influenced by the way the intrinsic capacity domains were operationalised in the study. We used a modified ICOPE Step 1 screener, and potential decline within the various domains was self-reported and not verified through objective testing. The high proportion reporting to be unable to rise from a chair five times in 14 s (locomotion domain, 23%) seems somewhat excessive. As the participants did not actually perform the task, and it may be one that few respondents have previously reflected upon, responses may be subject to random error. It is not unlikely that objective testing would have produced lower estimates. In contrast, the proportion of patients reporting cognitive decline (6%) was substantially lower than national prevalence estimates on cognitive impairment (dementia 15% and mild cognitive impairment 36% among adults ≥ 70 years) [23]. This may also be attributable to the use of self-reporting rather than objective tests, as self-reported cognitive problems require insight and are known to have lower sensitivity than objective tests [24]. Also, the data collection method may have favored cognitively healthier individuals, as respondents had to be able to complete the paper-based survey. When interpreting the findings regarding prevalence and what domains were most strongly associated with poor self-rated health, it should be kept in mind that the thresholds for defining decline vary across domains and may not represent equivalent levels of impairment. Accordingly, prevalence estimates and observed associations should be interpreted with appropriate caution.

### Contextualisation with existing literature

The overall prevalence of intrinsic capacity decline detected in our study (60%) aligns well with pooled estimates from ICOPE Step 1 screenings of community-dwelling adults aged ≥ 60 years globally (55%) [25]. A scoping review published in 2023 aimed to summarise the ability of ICOPE Step 1 to detect impairments confirmed through in-depth tests [26]. The authors found only one peer-reviewed article reporting relevant findings, in which the overall sensitivity was 95%, while the domain-specific sensitivity ranged from 75% (cognitive decline) to 100% (malnutrition and hearing loss) [27]. Specificity ranged from 74% (depressive symptoms) to 93% (limited mobility).

Direct comparisons of domain-specific prevalence estimates are futile given differences in settings, assessment methods and inclusion criteria. Nevertheless, the observed high prevalences of decline within the sensory and locomotion domains are noteworthy, given established evidence linking sensorimotor impairment to adverse health outcomes, specifically falls and dementia [28,29]. Falls and dementia both have substantial impact on the independence and quality of life of older adults, and the conditions represent major cost-burdens for the Norwegian healthcare system, particularly through increased demand for long-term care [30].

Our study showed that intrinsic capacity decline increased with increasing age, mirroring literature from other regions [31–33]. Furthermore, we found decline to be more prevalent among individuals with lower socioeconomic status, which is also consistent with findings from other regions [31–33]. The relationship between intrinsic capacity and poor self-rated health, which was found to be particularly strong for the psychological well-being and locomotion domains, is also in accordance with previous research from other countries [34,35]. The link between depressive symptoms and poor self-rated health observed here and elsewhere is unsurprising, as such symptoms directly shape subjective evaluations of health. While previous studies have found intrinsic capacity to be more common among females across domains, only visual impairment was associated with female sex in our study [31,32]. The reason for this discrepancy is unclear.

### Strengths and limitations

As the first study of intrinsic capacity in this setting, our findings provide important insight into the ageing population in general practice in Norway and offer supportive evidence for the relevance of the ICOPE framework in a new context. The broad recruitment, without exclusion based on reason for GP visit, high response rate and large sample size, with patients recruited from a wide range of practices in rural and urban settings, represents strengths of our study. As the items for assessing intrinsic capacity focused mainly on patients’ current situation, recall bias is expected to be low. However, our study also has some important limitations. The cross-sectional design prevents conclusions about causality, and there are concerns regarding representativity. Given recruitment from general practice waiting rooms, where individuals with frequent GP attendance were more likely to be included, the study population would be expected to have poorer overall health than the general older population. However, the data collection process may have contributed to a selection bias towards healthier patients, as students may have been reluctant to invite patients with prominent signs of disability, anticipating difficulties in completing the survey. Compared to the older population in Norway, a higher proportion had completed higher education among the included patients [32]. Hence, the results may reflect a relatively resourceful group within the target population, and the prevalence of intrinsic capacity decline may be underestimated. As the survey was only available in Norwegian, patients born elsewhere were bound to be underrepresented in our sample (4% vs. 8% in the general older population) [36,37]. All data were self-reported and collected at a single time point, thus, the findings may be subject to variability in interpretation of items and common method bias. Lastly, important confounders may not be captured in the limited dataset.

### Implications

Our results sit within a broader discussion of how primary care should respond to an increasing number of older adults, coinciding with an emerging shortage of health care personnel [38,39]. By capturing early decline in function and providing suggestions for evidence-based interventions, the intrinsic capacity framework offers a potentially useful complement to traditional disease-oriented approaches and may aid in identifying vulnerabilities before they manifest as frailty and disability [2,9]. The high prevalence of decline observed in our study lends empirical support to addressing intrinsic capacity in Norwegian general practice. However, whether ICOPE represents a feasible and appropriate approach in Norwegian primary care warrants further investigations. Implementation would need to take into account the resources required to support assessment and subsequent interventions, as well as follow-up capacity and opportunity costs in primary care.

Nevertheless, projections indicate a marked increase in the number of older adults living with frailty in Norway over the coming decades, thus, efforts to prevent or postpone functional decline should be a priority for healthcare services [40]. The longitudinal and coordinating role of GPs in Norway places them in a favorable position to recognise, monitor and respond to age-related changes in function. Notably, many of the assessment and intervention strategies proposed within the ICOPE framework align with existing national guidelines and areas of responsibility for the GPs in Norway, including recommendations for assessment and follow-up of patients with suspected dementia, prevention and management of malnutrition, falls prevention and mental health care for older adults [41–44]. In light of previous research linking sensorimotor impairment to adverse outcomes, our findings particularly reinforce the importance of adhering to the guideline on falls prevention for promoting healthy ageing [42]. This guideline target impairments within the sensorimotor domains, and incorporate exercise interventions that align with approaches shown to preserve function and delay the onset of frailty [10,42]. Sensorimotor impairment is increasingly recognised as a modifiable risk factor for dementia, further underscoring the importance of preserving function within these domains [45]. Our observations further suggest that decline in intrinsic capacity disproportionately affects socioeconomically disadvantaged individuals, placing them at increased risk of frailty and loss of independence. Previous research has shown that socioeconomically disadvantaged older adults are less likely to seek care for early functional changes [46]. GPs should therefore be aware that emerging decline may be more likely to go unrecognised in this group.

## Conclusions

Intrinsic capacity decline was common among older adults attending Norwegian general practice, especially among socioeconomically disadvantaged individuals, and was strongly associated with poor self-rated health. These findings may help GPs identify older patients who are at increased risk of adverse health trajectories. The ICOPE framework may have a role in supporting healthy ageing, although further research is needed to determine whether and how ICOPE should be integrated into general practice in Norway.

## Supplementary Material

Supplementary file 1_Intrinsic capacity GP Norway.docx

## Data Availability

Data are available from the corresponding author on reasonable request.
