# Supplementary material for: Intrinsic capacity decline and self-rated health in patients ≥ 65 years: a survey in general practice waiting rooms in Norway
Source: Scand J Prim Health Care. 2026 Oct 27;44(1):2748805. doi: 10.1080/02813432.2026.2748805 (PMC13715328; doi:10.1080/02813432.2026.2748805)
Supplement: Supplementary file 1 [file IPRI_A_2748805_SM6193.docx]

Supplementary material for Intrinsic capacity decline and self-rated health in patients ≥ 65 years: A survey in general practice waiting rooms in Norway. Scand J Prim Health Care 2026. Sebjørnsen I, Bakken MS, Hjörleifsson S, Hetlevik Ø, Bondevik GT, Berg-Utby C, Skonnord T, Brænd AM, Romøren M.

**Supplementary table 1:** Results from age-stratified Poisson regression analyses for association between declines in intrinsic capacity and poor self-rated heath, adjusted for sex, educational level, birthplace and economy. Associations are reported as relative risks (RR) with 95% confidence intervals (CI).

| **Age (years)** | **Any** | **Cognitive decline** | **Limited mobility** | **Malnutrition** | **Hearing loss** | **Visual impairment** | **Depressive symptoms** |
| --- | --- | --- | --- | --- | --- | --- | --- |
| **65-74** | 4.55 (2.75-7.55) | 2.78 (1.66-4.65) | 3.15 (2.20-4.50) | 1.73 (1.17-2.55) | 1.46 (1.02-2.10) | 1.59 (1.07-2.37) | 6.06 (4.26-8.63) |
| **75-84** | 4.40 (2.31-8.39) | 2.13 (1.27-3.56) | 3.95 (2.67-5.84) | 2.12 (1.44-3.13) | 1.32 (0.90-1.93) | 1.82 (1.22-2.73) | 4.64 (3.21-6.71) |
| **≥ 85** | 4.85 (0.66-35.72) | 2.07 (0.97-4.42) | 2.93 (1.19-7.24) | 2.93 (1.28-6.67) | 2.45 (1.07-5.63) | 1.97 (0.90-4.31) | 4.53 (2.18-9.40) |

**Supplementary table 2:** Results from sex-stratified Poisson regression analyses for association between declines in intrinsic capacity and poor self-rated heath, adjusted for age, educational level, birthplace and economy. Associations are reported as relative risks (RR) with 95% confidence intervals (CI).

| **Sex** | **Any** | **Cognitive decline** | **Limited mobility** | **Malnutrition** | **Hearing loss** | **Visual impairment** | **Depressive symptoms** |
| --- | --- | --- | --- | --- | --- | --- | --- |
| **Female** | 4.74 (2.73-8.23) | 1.55 (0.85-2.83) | 3.47 (2.47-4.88) | 1.76 (1.22-2.54) | 1.45 (1.03-2.03) | 1.71 (1.19-2.44) | 5.26 (3.78-7.32) |
| **Male** | 4.18 (2.40-7.26) | 2.99 (1.96-4.57) | 3.28 (2.25-4.76) | 2.22 (1.54-3.20) | 1.50 (1.05-2.14) | 1.67 (1.12-2.48) | 5.03 (3.53-7.16) |

**Supplementary figure 1: Self-rated health by status of intrinsic capacity (overall and by domain)**

|  | Very good | Fairly good | Neither good nor poor | Poor |
| --- | --- | --- | --- | --- |
| Any IC decline | 6 | 42 | 35 | 17 |
| No IC decline | 15 | 58 | 23 | 3 |
| Cognitive decline | 4 | 28 | 38 | 30 |
| No cognitive decline | 10 | 49 | 30 | 11 |
| Limited mobility | 4 | 31 | 40 | 26 |
| No limited mobility | 12 | 53 | 27 | 7 |
| Malnutrition | 5 | 37 | 37 | 21 |
| No malnutrition | 11 | 51 | 28 | 9 |
| Visual impairment | 5 | 40 | 36 | 19 |
| No visual impairment | 11 | 50 | 29 | 10 |
| Hearing loss | 7 | 44 | 33 | 16 |
| No hearing loss | 11 | 50 | 29 | 10 |
| Depressive symptoms | 3 | 24 | 26 | 47 |
| No depressive symptoms | 11 | 51 | 30 | 8 |
